# Vasculopathy in transthyretin Val30Met familial amyloid polyneuropathy

**DOI:** 10.1186/1750-1172-10-S1-O11

**Published:** 2015-11-02

**Authors:** Haruki Koike, Shohei Ikeda, Mie Takahashi, Yuichi Kawagashira, Masahiro Iijima, Masahisa Katsuno, Gen Sobue

**Affiliations:** 1 Nagoya University Graduate School of Medicine, Neurology, 466-8550, Nagoya, Japan

## Background

Transthyretin (TTR) Val30Met-associated familial amyloid polyneuropathy (FAP ATTR Val30Met) is the most common form of FAP and has become prevalent in areas other than conventional endemic foci. The clinicopathological features of FAP ATTR Val30Met are known to vary between endemic foci and non-endemic areas in Japan. Characteristic features of early-onset cases from Japanese endemic foci include the presence of sensory dissociation and marked autonomic dysfunction associated with a predominant loss of small-diameter myelinated and unmyelinated nerve fibers. These characteristics are uncommon in late-onset cases from non-endemic areas.

## Methods

We examined sural nerve biopsy specimens from 42 patients with FAP ATTR Val30Met using electron microscopy, particularly focusing on the morphology of nerve microvascular endothelial cells. This study included 5 early-onset cases from endemic foci (3 men and 2 women) and 37 late-onset cases from non-endemic areas (33 men and 4 women).

## Results

Greater amyloid deposition was observed in early-onset cases than in late-onset cases, whereas reduced nerve fiber density was more conspicuous in late-onset cases than in early-onset cases. Retraction of the processes of Schwann cells associated with amyloid fibrils was observed, particularly in early-onset cases. In addition, basement and cytoplasmic membranes of Schwann cells associated with amyloid fibrils were indistinct, indicating that direct invasion of amyloid to Schwann cells had resulted in predominantly small-fiber axonal loss characteristic of early-onset cases. In late-onset cases, nerve microvascular endothelial cell morphology was frequently found to be abnormal with loss of tight junctions and fenestrations.

## Conclusion

These findings suggest that, in addition to direct invasion of amyloid to Schwann cells, the disruption of the blood–nerve barriers contributes to the pathogenesis of neuropathy in FAP.

